# Transient ischemic attack: an unusual presentation of a carotid body tumor^☆^

**DOI:** 10.1016/j.bjorl.2016.02.002

**Published:** 2016-04-13

**Authors:** Mojtaba Maleki, Mahdi Safdarian, Ali Daneshvar

**Affiliations:** Iran University of Medical Sciences, Rasoul Akram Hospital, Tehran, Iran

## Introduction

Carotid body tumors (CBTs) are the most common paragangliomas, with an incidence of one in every 30,000 people in the general population.^1^

They usually affect middle-aged patients with chronic hypoxia and present as a cervical mass with lower cranial nerve palsies. CBTs are rarely malignant, usually asymptomatic, and have generally been associated with substantive procedural complications, especially blood loss.^2^

Surgical resection is recommended for all CBTs in healthy patients due to the risk of local complications related to tumor size and a small but definite risk of malignancy.^3^, ^4^ Despite progress in CBT imaging and surgical techniques, cranial nerve deficit, stroke, and death continue to affect 10–40% of patients undergoing curative surgical resection.

While most CBT complications occur during or post-surgery, CBTs should be considered among the uncommon causes of Transient Ischemic Attacks (TIAs) and stroke.^5^ Here we present a case of carotid body tumor in a patient with a history of TIA one month earlier.

## Case report

**Figure 1:**
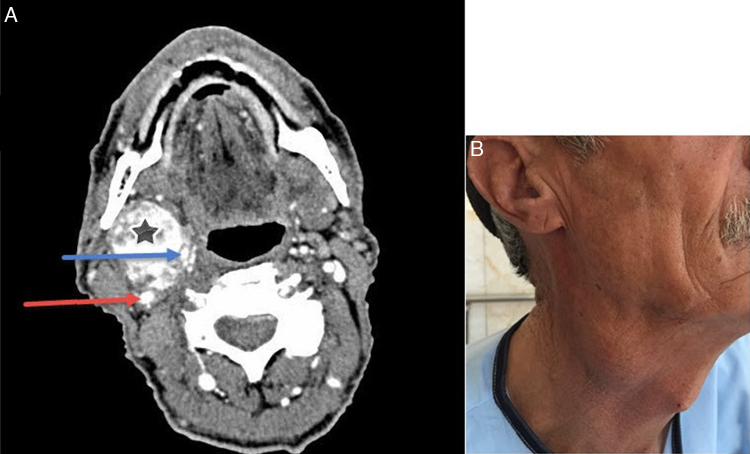
(A) Spiral neck CT scan with IV contrast shows CBT (star), ICA (red arrow) and ECA (blue arrow). (B) A 60 year-old man with a painless, non-pulsatile right neck mass since 40 years ago.

A 60 year-old man referred to the otolaryngology clinic of the Firoozgar hospital complaining of a painless, pulsatile right neck mass with a gradual growth since 40 years ago (Fig. 1).

He had no complaint of dysphagia or odynophagia, voice change and aspiration or nasal obstruction, and the upper limb muscular force was normal. No history of infection or rapid growth was found.

The patient reported a history of TIA starting less than 1 month earlier, presenting with left side upper limb weakness which was gradually getting better and resolved completely within days. The patient was not hypertensive or diabetic. Other etiologies of stroke were excluded. In the physical examination, a non-tender solid mass measuring 4 cm × 4 cm was found on the right side, level II–III of the neck with a thrill on palpation. The mass was mobile in horizontal vector but fixed in vertical movement (positive Fontaine sign). In systemic evaluation, no LAP was found in the cervical region, and neurologic examinations showed no cranial nerve deficit; normal force was detected in both side limbs.

Complete blood count, electrolytes, lipids and cholesterol level were normal. Electrocardiography showed no cardiac arrhythmia or abnormality. The sonography reported normal ICA (Internal Carotid Artery) and CCAs’ (Common Carotid Arteries) flow on both sides and a heterogeneous mass with high internal vascularity in the right carotid artery bifurcation. Spiral brain CT scan without contrast demonstrated a focal hypodense lesion in the anterior limb of the right internal capsule, indicating a lacunar infarct, and a mild hypodensity in the right parietal lobe, suggesting an old ischemia.

In the cervical CT scan, a heterogeneous 39 mm × 40 mm × 62 mm solid mass was detected in the right carotid artery bifurcation, from the oropharynx level to the supraglot, splitting the carotid artery branches with a significant enhancement.

Transcranial Color-Coded duplex Sonography (TCCS) reported normal anterior and posterior Peak Systolic Velocities (PSVs) and Mean Flow Velocities (MFVs). Normal flow velocity and direction in right and left ECAs (External Carotid Arteries), ICAs and CCAs with increased right and left CCAs Intima-Media Thickness (IMT). A vascularized mass-like lesion was also reported inside the carotid artery bifurcation (suggesting carotid body tumor) in addition to grade B (0–15%) stenosis in left and right ICAs.

Digital Subtraction Angiography (DSA) of carotids and brain was done with the indication of right-sided vascular neck mass, suggesting carotid body tumor. At right CCA, ICA and ECA selective injection, there was a tumoral low vascular blush on right CCA bifurcation that displaced ICA to posterolateral without invasion and was supplied by multiple fine branches from right ECA.

## Discussion

Although it is a rare neoplasm, CBT should be included in the differential diagnosis of any patient with an anterior lateral neck mass. Physical findings that support the diagnosis include a non-expansible pulsatile mass with the ability to move from side to side but not vertically.^6^ CBT usually presents as a painless, slow-growing, compressible mass in the upper neck.^1^ Local discomfort, recent increase in size, headache, dizziness, dysphagia, voice and hearing changes are other symptoms suggesting a functionally active tumor.^5^

The diagnosis is supported further by radiological procedures including sonography, CT scan, MRI and arteriography. Head and neck CT angiography also provides excellent tumor characterization, defines the superior and medial extent of the tumor, identifies concomitant paragangliomas, and allows for tumor volume assessment.^1^

The cerebrovascular accidents cited in literature are postoperative and also reported as one of the main causes of death. In our patient, an ischemic mechanism appears to be the most probable explanation of the symptoms. The absence of solid evidence of atherosclerotic changes on angiography makes other possibilities such as embolism related to CBT rather improbable.

## Conclusion

Considering the insistent growth of CBT, enclosing vital neurovascular structures, and the significant incidence of malignancy, surgical excision is the treatment of choice. Even though cervical CT scan is very useful for studying CBTs, this exploration does not exclude the need for angiography.

## Informed patient consent

The patient has consented to the submission of the case report for submission to the journal.

## Conflicts of interest

The authors declare no conflicts of interest.

## References

[1] Power A.H., Bower T.C., Kasperbauer J., Link M.J., Oderich G., Cloft H. (2012). Impact of preoperative embolization on outcomes of carotid body tumor resections. J Vasc Surg.

[2] Rao A.B., Koeller K.K., Adair C.F., From the archives of the AFIP (1999). Paragangliomas of the head and neck: radiologic–pathologic correlation. Radiographics.

[3] Nora J.D., Hallett J.W., O’Brien P.C., Naessens J.M., Cherry K.J., Pairolero P.C. (1988). Surgical resection of carotid body tumors: long-term survival, recurrence, and metastasis. Mayo Clin Proc.

[4] Hallett J.W., Nora J.D., Hollier L.H., Cherry K.J., Pairolero P.C. (1988). Trends in neurovascular complications of surgical management for carotid body and cervical paragangliomas: a fifty-year experience with 153 tumors. J Vasc Surg.

[5] Chamorro Sanchez A., Varela de Seijas E., Matesanz Matesanz J., Trapero V.L. (1988). Carotid body tumor: unusual cause of transient ischemic attacks. Stroke.

[6] Amsalu A., Anderson B., Tesfaye W. (2013). Giant malignant carotid body tumor in a 40 years old woman: a case report from Gondar University Hospital. Ethiop Med J.

